# Does the rubella immunoglobulin G affect the severity of COVID- 19? Rubella immunoglobulin G and COVID- 19

**DOI:** 10.1186/s12866-022-02563-5

**Published:** 2022-06-11

**Authors:** Leyla Sahebi, Mohsen Hosseini, Alireza Abdollahi, Nahid Farrokhzad, Samrand Fattah Ghazi, Farzaneh Samaei Noroozi, Fereshteh Ghiasvand

**Affiliations:** 1 Maternal, Fetal and Neonatal Research Center, Family Health Research Institute, Tehran University of Medical Science, Tehran, Iran; 2 School of Medicine, Tehran University of Medical Science, Tehran, Iran; 3 Department of Pathology, School of Medicine, Imam Khomeini Hospital Complex, Tehran University of Medical Sciences, Tehran, Iran; 4 Department of Anesthesiology and Critical Care, Imam Khomeini Hospital Complex, Tehran University of Medical Sciences, Tehran, Iran; 5 Central Laboratory, Imam Khomeini Hospital Complex, Tehran University of Medical Sciences, Tehran, Iran; 6 Department of Infectious Diseases, Tehran University of Medical Sciences, Tehran, Iran

**Keywords:** COVID-19, Immunoglobulin G, Rubella

## Abstract

**Objective & aim:**

The coronavirus disease, so far (COVID-19) has brought about millions of infections and fatalities throughout the world. Our aim was to determine the correlation between rubella IGG titers with the severity COVID-19.

**Materials & methods:**

This study was conducted among COVID-19 confirmed patients over 18 years of age. The disease severity levels were categorized by WHO interim guidance. The rubella-specific IgG antibody-titer spectrum was measured (within first 48 h of hospitalization) by enzyme-linked immunosorbent assay (ELISA).

**Result:**

In a study of 46 inpatients with varying COVID-19 disease severity (mild, moderate, severe, and critical), we observed a negative correlation between rubella IgG antibody titers and COVID‐19 severity (*P*-Value = 0.017), There was an interaction between COVID-19 vaccination history and rubella IGG on severity COVID-19 (*P*-Value = 0.0015). There was an interaction between age group under 44 years (including national measles- rubella (MR) vaccination in Iran) and rubella IGG titers on severity COVID-19 too (*p*-value = 0.014).

**Conclusion:**

In conclusion, MR vaccination seems to have a positive effect in reducing the severity of the disease, emphasizing that, the important and separate effect of the IGG rubella (due to natural or extrinsic immunity) titers is determining.

## Introduction

The present pandemic of coronavirus disease (COVID-19) has been creating unprecedented crises all around the world. COVID-19 is caused by severe acute respiratory syndrome coronavirus 2 (SARS-CoV-2) virus strain [1]. There are still many ambiguities about this disease. COVID-19 has severely affected some countries and spared others [2]. Overall, fatality ratio of COVID-19 disease is estimated from 0.1% (Burundi country; in Africa) to 19.4% (Yemen country; in Asia). COVID-19 mortality is approximately 2% in Iran [3]. The severity and mortality of COVID-19 has been widely reported to be influenced by age, sex and underlying comorbidities [4].

The disease is highly contagious, and no standard treatment for SARS-CoV-2 is currently available; several clinical trials are investigating repurposed drugs [1].

COVID-19 has presented various paradoxes that, if scientific justification can be found, may provide clues to controlling the pandemic in addition to universal COVID-19 vaccination. (1) Young children are mostly protected from severe disease. (2) numerous countries have less than 1% mortality (as New-Zealand, Maldives, Qatar, Singapore, Norway, Cuba …) (3) lots of people, despite prolonged close contact with infected patients, never test positive and, (4) nearly 50% of people who test positive for COVID-19 are asymptomatic (5).

Some researchers have theorized that the measles-mumps-rubella (MMR) vaccine may play a decisive role in these disparities [1, 5–10]. There are reports that appear to suggest that several currently available vaccines (including polio, Haemophilus influenzae type-B, MMR, and pneumococcal) may offer significant protection against COVID-19 via a nonspecific immunity [8, 11, 12]. However, these findings may actually be reflecting a response to MMR vaccination, which is often administered in conjunction with others [5, 9, 13].

The MMR vaccine was introduced in 1971. It was most commonly given as a single vaccination from 1971–1978 and two -dose vaccinations of 1979. Thus, most people 42–49 years would likely have had at least one MMR vaccination and those 41 years and younger two MMR vaccinations. This vaccine history may be a probable reason for a COVID-19 death rate pivot point close to age 50. The fact that people aged 40–49 has a marginally higher death rate than those under 40, may be due to the fact that they have received only a single (measles-rubella containing vaccines) MRCV dose [14]. It seems that countries with vaccination programs established in recent decades( like Italy, France, and United kingdom) appear to show the lowest incidence of death from COVID-19 [14] National measles–rubella vaccination campaign was performed in December 2003 in Iran on aged 5–25 years (people under 44 years old at the end of 2021 [15].

In general, researchers have had different views on the relationship between MRCV and COVID -19 disease; some of them have mentioned the cumulative and nonspecific effect of vaccines reduces the severity of the disease [1, 11, 16, 17].

Some others have documented the independent effect of a specific vaccine such as measles, mumps or rubella or even bacille Calmette-Guerin (BCG) on severity and fatality of COVID-19 disease [5, 17–20], and some other researchers have generally rejected the effect of live vaccines other than the specific COVID-19 vaccine [21–23].

There are many ambiguities in this regard, while so far the majority of limited studies have evaluated the effectiveness of the MRCV, identifying the immune pattern (nonspecific or specific) created against to Coronavirus, and its use in disease management can help eliminate the disease.

In this study, we aimed to investigate the correlation between rubella immunoglobulin G (IGG) titer and the severity of COVID-19 disease and role of COVID-19 vaccine in this correlation regardless of MRCV history.

## Method

This was a cross-sectional study and sampling was conducted prospectively, from September, 2021, to December, 2021. All patients were recruited from Imam Khomeini Hospital complex (IKHC) Tehran (capital of Iran).Informed consent was obtained from all subjects. This study was approved by the Ethics Committee (IR.TUMS.IKHC.REC.1400.112). All methods were performed in accordance with the declarations of Helsinki.

The target sample size of 46 was calculated assuming a 0.42 correlation between rubella IGG titer and COVID-19 severity, 5% probability of type I error, and 20% probability of type II error. (Based on the proportional allocation method; 10 patients from intensive care units (ICU) and 36 patients from Corona ward were selected.

All patients over 18 years of age whose COVID- 19 disease were confirmed by real-time polymerase chain reaction (RT-PCR) or by chest computerized tomography (CT) (diagnosed according to WHO interim guidance) were included to the study. Exclusion criteria were those who were unwilling to participate in study. Demographic information (age, gender, smoking history), clinical (included history of chronic disease (Chronic Obstructive Pulmonary Disease (COPD), asthma, Cardiovascular Diseases (CVD), renal disease, hypertension, diabetes, malignancies, and immunodeficiency), para-clinical characteristics (Red Blood Cell (RBC), Hemoglobin(HB), Hematocrit (HCT), White Blood Cell (WBC), Platelet, Erythrocyte Sedimentation Rate(ESR), C-Reactive Protein(CRP), Aspartate Aminotransferase (AST), Alanine Aminotransferase(ALT), Creatine (Cr), lactate dehydrogenase(LDH), Creatine Phosphokinase (CPK), Troponin,and Ddimer), Immunoglobulin G (IGG) rubella (within first 48 h of hospitalization, the history of COVID-19 vaccination and clinical symptoms and hospitalization outcome of COVID-19 were documented. The rubella-specific IgG antibody-titer spectrum was measured by enzyme-linked immunosorbent assay (ELISA).(The measurement was performed by an experienced technician and a device) The disease severity levels were as follows: mild COVID-19 defined as symptomatic disease without evidence of viral pneumonia or hypoxia, moderate COVID-19 defined as clinical signs of pneumonia (fever(> 38° C), cough, dyspnea, fast breathing), SpO_2_ between 90 and 93%, on room air and pulmonary involvement less than 50%. Severe COVID-19 defined as clinical signs of pneumonia (fever, cough, dyspnea, and fast breathing plus 1 of the following: respiratory rate > 30 breaths per minute, severe respiratory distress, SpO2 < 90% on room air, or pulmonary involvement more than 50% of the lung in CT scan. Critical COVID-19 is defined as presence of at least one of the following: the onset of symptoms of respiratory failure despite non-invasive oxygen therapy, septic shock, and multiple organ dysfunctions [24].

Analysis: Descriptive data were presented as mean (± SD) for normally distributed continuous variables and as median (± IQR) for non-normally distributed data. Categorical variables were presented as percentages. Spearman correlation was tested for association in nonparametric variables. Multiple ordinal regression analysis was modeled for determination of interaction between variables. Two and multiple (more than two) independent samples were tested by the Mann Whitney u test and Kruskal–Wallis rank-sum test respectively. The χ^2^ test was performed to compare count data. A 2-tailed value of *P* < 0.05 was considered statistically significant. All statistical analyses were performed using SPSS version 20 (SPSS, Chicago, IL).

## Result

Among the 46 patients with confirmed COVID-19 positive, the mean age (SD) was 50.78 (16.02) years and 19 out of the 46 patients (41.3%) were male. Of the 46 patients, 23 cases had comorbidities, including 14 hypertension (30.4%), 8 heart failure (17.4%), 6 diabetes mellitus (13.0%), and one cancer (2.2%). The most common clinical symptoms of COVID- 19 were cough (63.0%), dyspnea (54.3%) and weakness (60.9%) respectively. Frequency (%) of oxygen therapy, needed intubation, and ventilation was 35(76.5%), 2(4.3%), and 2(4.3%), respectively (Table 1).

**Table 1:** Demographic, clinical and para clinical information of the subjects

Variables	Cofactors	n (%)	Variables	Cofactors	n (%)
Age (years) Mean, SD		50.78(16.02)	Biochemical parameters collected on the day of admission, mean ± SD^d^	WBC *mean* ± *SD*	9.17(5.3)
Gender	Male	19(41.3)		RBC *mean* ± *SD*	4.61(0.93)
	Female	27(58.7)		HB *mean* ± *SD*	12.73(1.96)
Inpatient ward	COVID ward	36(78.3)		HCT *mean* ± *SD*	38.23(4.85)
	ICU	10(21.4)		platelet *mean* ± *SD*	240.48(118.9)
COVID vaccination^a^	Yes	23(50.0)		ESR *mean* ± *SD*	52.03(25.05)
	No	23(50.0)		CRP *mean* ± *SD*	81.16(51.15)
Smoking history	Yes	2(4.3)		AST *mean* ± *SD*	54.11(36.35)
	No	44(95.7)		ALT *mean* ± *SD*	55.91(59.78)
Medical history	Hypertension	14(30.4)		Cr *mean* ± *SD*	0.95(0.39)
	Heart failure	8(17.4)		LDH *mean* ± *SD*	719.09(254.9)
	Diabetes mellitus	6(13.0)		CPK *mean* ± *SD*	230.04(342.8)
	Immuno-deficiency^b^	2(4.3)		Troponin *mean* ± *SD*	0.64(2.64)
	Cancer	1((2.2)		D dimer *mean* ± *SD*	1068.64(901.69)
	Respiratory disease^c^	0(0.0)	O2saturation *mean* ± *SD*		87.71(7.6)
Clinical symptoms	cough	29(63.0)	Temperature *mean* ± *SD*		36.77(0.36)
	Weakness/ Fatigue	28(60.9)	Respiratory rate *mean* ± *SD*		19.82(5.5)
	dyspnea	25(54.3)	Blood Pressure (systolic) *mean* ± *SD*		11.59(1.36)
	fever	24(52.2)	Blood Pressure(diastolic) *mean* ± *SD*		7.14(1.1)
	chills	15(32.6)	ventilation		2(4.3%)
	headache	10(21.7)	intubation		2(4.3)
	myalgia	9(19.6)	Oxygen therapy		35(76.5)
	Sore throat	9(19.6)	lung_involv_40		8(17.4%)
	Chest pain	8(7.4)	lung_involv_60		18(39.1)
	Nausea/Vomiting	8(17.3)	lung_involv_80		14(30.4)
	Anorexia	6(13.0)	arrhythmia		1(4.3)
	Taste disorder	6(13.0)	STT change		1(17.4)
	Olfactory disorder	3(6.5)	Outcome	discharge	45(97.82)
	sputum	2(4.3)		expire	1(2.17)
	Confusion	2(4.3)			
	Diarrhea	1(2.2)			
	rhinitis	0(0.0)			

^a^At least one dose

^b^Corticosteroid therapy, chemotherapy, radiotherapy history, and Immune Deficiency Diseases

^c^COPD, asthma

^d^*RBC* Red blood cell, *HB* Hemoglobin, *HCT* Hematocrit, *ESR* Erythrocyte sedimentation rate, *CRP* C-reactive protein, *AST* Aspartate aminotransferase, *ALT* Alanine aminotransferase, *Cr* Creatine LDH, lactate dehydrogenase, *CPK* Creatine kinase

Totally 50.0% (23/46) of patients had history of COVID-19 vaccination. Booster dose of vaccination was 13.6%. The average (SD) of the last dose of vaccination was 5.35 (1.35) months (range = 2–5 months). The prescribed vaccine brand names were Sinopharm’s BBIBP-CorV (14/44), Gamaleya’s Sputnik V (2/44), Oxford-AstraZeneca’s ChAdOx1/AZD1222 (2/44) and COVIran Barekat(3/44). The brand of vaccine was not recorded in 2 patients.

The basic, clinical and para-clinical characteristics of the subjects are shown in Table 1. COVID‐19 severity was categorized as, mild (6/46, 13.0%), moderate (13/46, 28.3%), severe (23/46, 50%), and critical (4/46, 8.7%). Mortality in our study population was 2.17% (1/46). (This person was an 84-year-old man with no underlying disease, with a history of two doses of Oxford-AstraZeneca’s ChAdOx1/AZD1222 (The booster dose was injected 5 months before hospitalization) with SPO2 equal to 72 and rubella IGG equal to 10 AU/ml).

Within the first 48 h after the hospitalization, the mean (SD) and median (IQR) of rubella IgG level in patients were 76.95(72.17) and 39.95(135) AU/ml respectively.

**Fig. 1:**
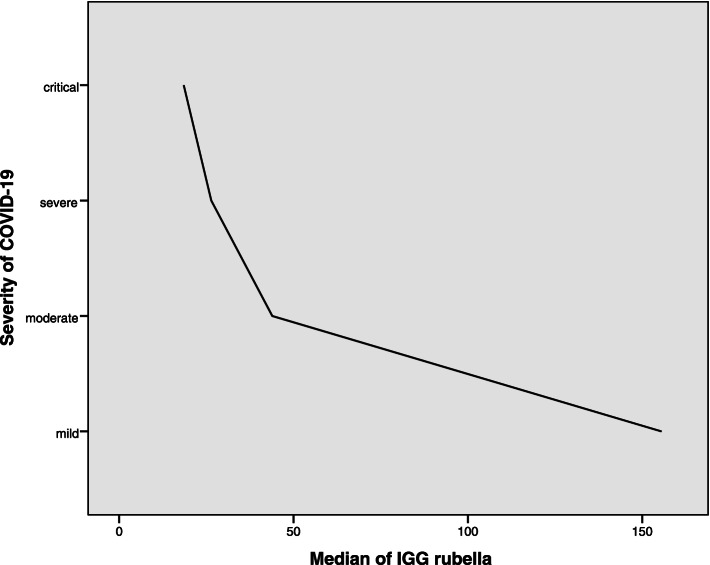
Correlation between the severity of COVID- 19 and rubella IGG levels

A moderate inverse correlation was observed between the severity of COVID- 19 and rubella IGG levels. (Spearman’s rho Correlation Coefficient = -0.365, *P*-value = 0.018) (Fig. 1). Also a moderate inverse correlation was found between SPO_2_ (%) level (< 94 and ≥ 94) and rubella IGG levels. (Spearman's rho Correlation Coefficient = -0.365, *P*-value = 0.018).

**Fig. 2:**
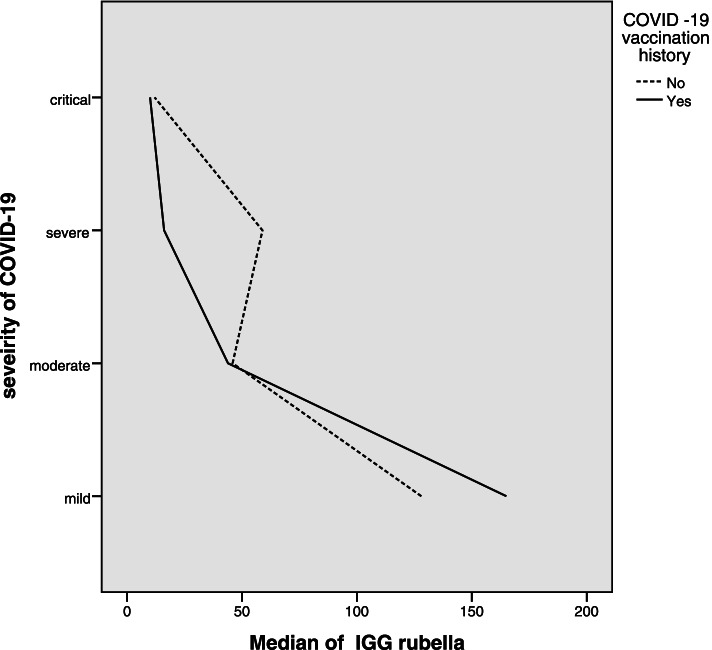
Correlation between severity of COVID-19 and rubella IGG in vaccinated and non-vaccinated group

We compared rubella IGG levels in the vaccinated and non-vaccinated groups. No statistically significant difference was observed between the two variables (Z Mann–Whitney U = -0.224, *P*-Value = 0.823). There was also no statistically significant difference between number vaccine dose (0, 1dose and 2 doses) and rubella IGG titer (X^2^ in Kruskal Wallis test = 5.4, *P*-Value = 0.067). We also analyzed correlation between severity of COVID-19 and rubella IGG separately in vaccinated and non- vaccinated groups. There was strong correlation between severity of COVID-19 and rubella IGG in vaccinated group (*r* = -0.615, *P*-Value = 0.002) (Fig. 2), but there was no significant correlation between severity of COVID-19 and rubella IGG in non-vaccinated group(*r* = -0.06, *P*-Value = 0.797).

There was a statistically significant difference between the median rubella IGG titer in the two age groups (equal and under 43 years and over 43 years (median (IQR) = 122(134), and 22.1 (88.0) respectively). The interaction between rubella IGG and COVID-19 vaccination history on severity of COVID- 19 was tested by multiple ordinal regression. There was an interaction between COVID-19 vaccinations (having vaccination) and IGG rubella titer on severity COVID-19. (*P*-Value for model fitting = 0.007, B = -0.018, *P*-Value = 0.03) (Table 2).

**Table 2:** Interaction between COVID-19 vaccinations (having vaccination) and IGG rubella on severity COVID-19 by ordinal regression analysis

	Variables		Estimate	Std. Error	*P*-Value
Threshold	Severity of COVID-19	Mild	-3.255	.853	.000
		Moderate	-1.179	.627	.060
		Severe	2.403	.871	.006
		Critical	-	-	-
Location	rubella IGG		-0.18	0.006	0.003
	COVID-19 vaccination	No	-0.426	0.896	0.635
		yes	-	-	-
	Interaction between independent variables	COVID-19 vaccination (no) * rubella IGG	-.001	.006	.903
		COVID-19 vaccination (yes) * rubella IGG	-.018	.006	.003

**Fig. 3:**
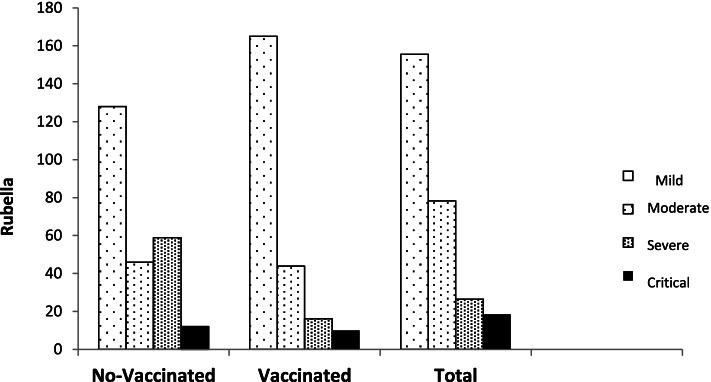
Distribution of rubella IGG level, based on different severities of the COVID-19 by vaccinated and unvaccinated groups

The median distribution of rubella IGG level, based on different severities of the COVID-19 disease and separately for vaccinated and unvaccinated groups, can be seen in Fig. 3.

We found inverse correlation between age, systolic blood pressure, ESR, platelet, and Cr with rubella IGG (*r* = -0.47, *P*-Value < 0.001; *r* = -0.42, *P*-Value = 0.007; *r*—0.32, *P*-Value = 0.034; and *r* = -0.312, *P*-Value = 0.042 respectively). But RBC and rubella IGG had positive correlation (*r* = 0.318, *P*-Value = 0.038). In the vaccinated group, variables of age, Cr, and LDH had inverse correlation with rubella IGG (*r* = -0.67, *P*-Value < 0.001; *r* = -0.504, *P*-Value = 0.02; and *r* = -0.486, *P*-Value = 0.048 respectively). In the unvaccinated group, systolic blood pressure, and ESR had inverse, and LDH had positive correlation with rubella IGG titers(*r* = -0.55; *P*-Value = 0.015; *r* = -0.535, *P*-Value = 0.027; and *r* = 0.54, *P*-Value = 0.02 respectively). Also we found an interaction between age ≤ 43 years and rubella IGG titer in severity of COVID-19 (*P*-Value for model fitting = 0.04, B = -0.016, *P*-Value = 0.026). There is an interaction between birth year and rubella IGG titers on severity COVID-19 too (*P*-Value for model fitting = 0.005, B = -0.002, *P*-Value = 0.014).

Comparison of some clinical and para-clinical factors with rubella IGG based on COVID-19 vaccination history is shown in Table 3.

**Table 3:** Comparison of some clinical and para clinical factors with rubella IGG based on COVID-19 vaccination history

Variables	Total			With history of COVID vaccination		Without history of COVID vaccination	
	Cofactors (code)	Median (IQR)	r(*P*-Value)	Median (IQR)	r(*P*-Value)	Median (IQR)	r(*P*-value)
Gender	Male(1)	36(101)	-0.043(0.775)	31.2(84)	0.033(0.882)	84.5(0.144)	-0.183(0.428)
	Female(2)	58.9(151)		90.5(0.157)		26.5(120)	
Inpatient ward	COVID ward(1)	64.45(143)	-0.197(0.19)	43.9(155)	-0.182(0.406)	70(125)	-0.25(0.274)
	ICU(2)	23.6(62)		24.3(35)		10.5(149)	
Underlying disease history	No(0)	111(146)	-0.224(0.138)	134.5(178)	-0411(0.057)	78.95(0.122)	-0.032(0.892)
	Yes(1)	26.5(104)		25.1(55)		26.5(160)	
Dyspnea	No(0)	43.45(129)	0.141(0.362)	59.25(155)	0.193(0.391)	78.95(186)	0.12(0.614)
	Yes(1)	39.95(129)		31.2(114)		70(114)	
Age (years)			**-0.47(< 0.001)**		**-0.674(0.001)**		-0.179(0.439)
Respiratory rate			0.162(0.295)		-0.047(0.837)		0.385(0.094)
Blood Pressure(sys)			**-0.397(0.008)**		-0.301(0.163)		**-0.548(0.015)**
Blood pressure(dias)			0.214(0.163)		-0.11(0. 616)		-0.313(0.193)
WBC			-0.124(0.435)		-0.239(0.296)		0.054(0.828)
RBC			**0.318(0.038)**		0.37(0.099)		0.259(0.271)
HB			0.285(0.064)		0.344(0.127)		0.292(0.21)
HCT			0.256(0.162)		0.338(0.134)		0.205(0.401)
ESR			**-0.429(0.007)**		-0.310(0.184)		**-0.535(0.027)**
platelet			**-0.32(0.034)**		-0.375(0.094)		-0.325(0.15)
CRP			-0.231(0.127)		-0.007(0.974)		-0.389(0.82)
AST			0.026(0.868)		0.174(0.439)		-0.124(594)
ALT			0.116(0.446)		0.270(0.224)		-0.044(0.855)
Cr			**-0.312(0.042)**		**-0.504(0.02)**		-0.174(0.463)
LDH			0.90(0.607)		**-0.486(0.048)**		**0.541(0.02)**
CPK			0.237(0.254)		0.408(0.147)		0.091(0.79)
Troponin			**-0.38(0.032)**		-0.302(0.223)		0.450(0.102)
Ddimer			-0.011(0.476)		-0.137(0.555)		-0.168(0.493)

## Discussion

Our study is the first study that was performed on the correlation of rubella IGG and severity of COVID-19 disease. This study showed that high IGG levels can play an important role in the development of milder COVID-19.

In this study, having COVID-19 vaccination history was not associated with rubella IGG level. However, existence of COVID-vaccination had positive accumulating effect with rubella IGG titer on reducing the severity of the COVID-19 disease. All patients reported as far as they remember, had no history of Measles, Mumps, and Rubella (MMR) vaccination recently.

Routinely, MMR vaccination includes children 12 and 18 months and also if necessary for women who are planning to become pregnant in Iran. Furthermore, a national measles–rubella vaccination campaign was initiated in December 2003 in Iran, targeting individuals aged 5–25 years (people under 44 years old at the end of 2021) [15] We compared the median of rubella IGG titers in the two age groups (> 43 and ≤ 43 years old) and there was a huge difference in IGG rubella level between the two age groups (about 100 units). In addition, an interaction between the age group under 44 years and IGG rubella titer was detected in development of milder COVID-19. There was a positive interaction between birth year (quantitative) variable and rubella IGG on COVID-19 severity too. During aging, comorbidities with chronic diseases, and changes in the immune system increase the chance of COVID- 19 disease severity [25], Therefore, the modification between the year of birth and the IGG rubella titer on the severity of the disease can be justified.

Some studies have reported that the MMR vaccine may protect against or reduce the severity, hospitalization, or mortality of coronavirus disease 2019 infection [1, 5–10]. This theory was introduced by Gold et al. (2020), after observing that recent countries with large-scale MMR vaccination are associated with the fewest COVID-19 deaths [14].

SARS-CoV-2 is an encapsulated, single-stranded RNA virus, belonging to the genus *Betacoronavirus*, subgenus *Sarbecovirus*, from the family of *Coronaviridae*. Sharing approximately 80% similarity with SARS-CoV-1 and 96% similarity with bat coronavirus, the genome of SARS-CoV-2 encodes 4 structural proteins, including the spike (S), envelope (E), membrane (M), and nucleocapsid (NP), besides other non-structural proteins [26].

MMR vaccines consist of attenuated enveloped ssRNA viruses that have glycoprotein spikes, similar to SARS-CoV2. There is 32%, 31% and 33%

homology between the spike amino acid sequences of measles, mumps and rubella, respectively, with that of the SARS-CoV2 [9].

Researchers have proposed this theory that preexisting immunity against COVID-19 may be due to the cross-reactivity with other antigens, for example the ones resulting from previous immunizations [27].

It is a theory that one or more of the MMR components may be structurally similar to SARS-CoV epitopes recognized by the immune system and may contribute to cross-immunity [16]. In some of studies, measles vaccine has been introduced as an important and effective factor in this cross-reactivity [5, 17, 18].

Hassani and colleagues (2021) reported that measles vaccination trigger those B cells cross-reactive with SARS-CoV-2 antigens leading to production of increased levels of measles-specific IgG [17]. Epithelial cells also carry Fc receptor on their surface that can interact with IgG and transport them for intracellular disruption of viral replication [5, 18].

In a homologous study by Marakasova, and Baranovaa, a similarity between the receptor binding domain (RBD) of the surface glycoprotein of SARS-CoV-2 causing coronavirus disease 2019 and the measles fusion glycoprotein was found too [19].

But in contrast, analysis of Klimczak et al. (2021) demonstrated that there was a statistically significant similarity between the distributions of base substitution densities in rubella and in each of three filtered SARS-CoV-2 Mutation Annotation Formats (MAFs) as well as a similarity in several prevailing types of base substitutions [20].

According to Anbarasua and colleagues, even though COVID 19 is impacting all aspects of life, the interesting scenario is slight symptoms in the young; mainly in children under 10 years of age, of note this age group is the most susceptible to infectious diseases [1]. So, it seems the extensive pediatric MMR vaccination schedule is effective in their safety and widespread vaccination is effective in their safety against COVID-19 [1]. They agreed that mass neonatal vaccination with MMR vaccines globally might result in innate immune responses leading to induction of interferons (IFN)s and NK cells, thus offering non-specific immunity against COVID- 19 virus in children [1, 17]. In confirmation of this theory, it was reported that milder symptoms observed in the 955 sailors on the USS Roosevelt navy who tested positive for COVID-19 (only one hospitalization) may have been due to the fact that all US navy recruits were compulsorily vaccinated with MMR [11].

However, a limited number of researchers disagreed with cross-reactivity effect in live vaccines including MMR and BCG and they have emphasized that some countries like Iran and Latin American countries, e.g., Chile, Argentina, and Ecuador, maintain 90% vaccine coverage, which started BCG vaccination in 1985 or even earlier, still have high mortality from COVID-19 [21, 22]. Also since the other set of live vaccines (BCG, polio, rotavirus, and chickenpox) also are administered at less than 1 year of age, this could result in a cumulative effect [23]. In confirmation of the lack of MMR vaccination effect in the prevention of more severe COVID -19 diseases, Lundberg and colleagues (in a study on health care workers (2021) proposed that MMR-vaccination up to 2.5 years prior affords no considerable protective effect against COVID-19 infection. However, in sex-stratified analyses, recently MMR-vaccinated men had 57% vaccine effectiveness at preventing symptomatic disease (*P* = 0.006) [28].

In our study, analyses was based on the rubella IGG titer (not MMR vaccination), and its measurement was conducted in the first 24 to 48 h of hospitalization. Although patients who are hospitalized due to COVID-19 show relatively more severe cases or underlying diseases, hospitalized patients also have different groups in disease severity.

The high level of rubella IGG titers in the age group under 44 years (individuals included in the 2003 national MR vaccination) and the presence of strong interaction between the individuals under 44 years and the rubella IGG level on the milder of the COVID-19 disease, the possibility of vaccine cumulative effect (including mumps, polio, BCG, etc.) could be rejected.

On the other hand, the increase in the rubella IGG titer is not always due to vaccination, natural circulation of the virus as a natural booster has played a major role in increasing antibodies [15] and it is well known that vaccine-induced immunity is lower than that resulting from natural exposure to the wild virus, and there are indications that vaccine-induced rubella immunity declines over time among those who received a single dose of vaccine [29].

One of the limitations of the present study was the low sample size. It is recommended to perform predictive analysis and diagnostic accuracy in studies with larger sample size (above 100 cases).

## Conclusion

In conclusion, MR vaccination seems to have a positive effect in the occurrence of milder cases of coronavirus disease 2019, emphasizing that, the specific effect of the rubella IGG (natural or acquired immunity) titers is determining. COVID- 19 vaccination with higher rubella IGG titer, provoke reduction on the severity of the disease as well.

## Data Availability

The datasets used during the current study available from the corresponding author on reasonable request.

## References

[1] Anbarasu AAO, Ramaiah SAOX, Livingstone P (2020). Vaccine repurposing approach for preventing COVID 19: can MMR vaccines reduce morbidity and mortality?. Hum Vaccin Immunother.

[2] Ashford JW, Gold JE, Huenergardt MA, Katz RBA, Strand SE, Bolanos J (2021). MMR Vaccination: A Potential Strategy to Reduce Severity and Mortality of COVID-19 Illness. Am J Med.

[3] Mortality in the most affected countries [Electronic]. Corona virus resource center. Johns Hopkins University & Medicines. [last update: June 4, 2022] . Available from: https://coronavirus.jhu.edu/data/mortality.

[4] Estimating mortality from COVID-19[Electronic]. World health organization [cited 4 August 2020]. Available from: https://www.who.int/publications-detail-redirect/WHO-2019-nCoV-Sci-Brief-Mortality-2020.1.

[5] Gold JA-O, Baumgartl WH, Okyay RA, Licht WE, Fidel PLJA-O, Noverr MC, et al. Analysis of Measles-Mumps-Rubella (MMR) Titers of Recovered COVID-19 Patients. mBio. 2020;11(6):e02628-20. Published 2020 Nov 20. 10.1128/mBio.02628-20. 10.1128/mBio.02628-20 PMC7686805 33219096

[6] Elhusseiny KM, Abd-Elhay FA, Kamel MG (2020). Possible therapeutic agents for COVID-19: a comprehensive review. Expert Rev Anti Infect Ther.

[7] Meenakshisundaram R, Senthilkumaran S, Thirumalaikolundusubramanian P (2020). Protective effects of vaccinations and endemic infections on COVID-19: A hypothesis. Med Hypotheses.

[8] Pawlowski C, Puranik A, Bandi H, Venkatakrishnan AJ, Agarwal V, Kennedy R (2021). Exploratory analysis of immunization records highlights decreased SARS-CoV-2 rates in individuals with recent non-COVID-19 vaccinations. Sci Rep.

[9] Sidiq KR, Sabir DK, Ali SM, Kodzius R (2020). Does Early Childhood Vaccination Protect Against COVID-19?. Front Mol Biosci.

[10] Larenas-Linnemann DAO, Rodríguez-Monroy F (2021). Thirty-six COVID-19 cases preventively vaccinated with mumps-measles-rubella vaccine: All mild course. Allergy.

[11] Fidel Paul L, NoverrMairi C, Gilmore Michael S (2020). Could an Unrelated Live Attenuated Vaccine Serve as a Preventive Measure To Dampen Septic Inflammation Associated with COVID-19 Infection?. mBio.

[12] Root-Bernstein RAO (2020). Age and Location in Severity of COVID-19 Pathology: Do Lactoferrin and Pneumococcal Vaccination Explain Low Infant Mortality and Regional Differences?. Bioessays.

[13] Eubank S, Eckstrand I, Lewis B, Venkatramanan S, Marathe M, Barrett CL. Commentary on Ferguson, et al. "Impact of Non-Pharmaceutical Interventions (NPIs) to Reduce COVID-19 Mortality and Healthcare Demand". Bull Math Biol. 2020 8;82(4):52. 10.1007/s11538-020-00726-x. 10.1007/s11538-020-00726-x PMC7140590 32270376

[14] Ashford JW, Gold JE, Huenergardt MA, Katz RBA, Strand SE, Bolanos J (2021). MMR Vaccination: A Potential Strategy to Reduce Severity and Mortality of COVID-19 Illness. Am J Med..

[15] Shafayi A, Mohammadi A (2021). A Review on Rubella Vaccine: Iran (1975–2010). Arch Razi Inst..

[16] Shimizu K, Teshima A, Mase H. Measles and Rubella during COVID-19 Pandemic: Future Challenges in Japan. Int J Environ Res Public Health. 2020;18(1):9. 10.3390/ijerph18010009 PMC7792618 33374998

[17] Hassani D, Amiri MM, Maghsood F, Salimi V, Kardar GA, Barati O (2021). Does prior immunization with measles, mumps, and rubella vaccines contribute to the antibody response to COVID-19 antigens?. Iran J Immunol..

[18] Gold JA-O, Hurley DJ, Rada BA-O, Baumgartl WH, Tilley LP, Licht WE. Reply to Marakasova and Baranova, "MMR Vaccine and COVID-19: Measles Protein Homology May Contribute to Cross-Reactivity or to Complement Activation Protection". mBio. 2021;12(1). 10.1128/mBio.03682-20 PMC7858077 33531392

[19] Marakasova E, Baranova A, Pirofski LA (2021). MMR Vaccine and COVID-19: Measles Protein Homology May Contribute to Cross-Reactivity or to Complement Activation Protection. mBio.

[20] Klimczak LJ, Randall TA, Saini N, Li JL, Gordenin DA. Similarity between mutation spectra in hypermutated genomes of rubella virus and in SARS-CoV-2 genomes accumulated during the COVID-19 pandemic. Preprint. PLoS ONE. 15(10):e0237689. 10.1371/journal.pone.0237689. 10.1371/journal.pone.0237689 PMC7531822 33006981

[21] Kantor IN (2020). BCG versus COVID-19?. Medicina (B Aires).

[22] Macedo A, Febra C (2020). Relation between BCG coverage rate and COVID-19 infection worldwide. Med Hypotheses.

[23] Özdemir Ö, Lloyd Richard E, Gilmore Michael S (2020). Measles-Mumps-Rubella Vaccine and COVID-19 Relationship. mBio.

[24] Organization WH (2021). Living guidance for clinical management of COVID-19.

[25] Mueller AL, McNamara MS, Sinclair DA (2020). Why does COVID-19 disproportionately affect older people?. Aging (Albany NY).

[26] Pal M, Berhanu G, Desalegn C, Kandi V. Severe Acute Respiratory Syndrome Coronavirus-2 (SARS-CoV-2): An Update. Cureus. 2020;12(3):e7423. 10.7759/cureus.7423. 10.7759/cureus.7423 PMC7182166 32337143

[27] Shimizu KAO, Mossialos E (2020). Accountability and transparency are vital in a pandemic response. J Gen Fam Med.

[28] Lundberg L, Bygdell M, Stukat von Feilitzen G, Woxenius S, Ohlsson C, Kindblom JM (2021). Recent MMR vaccination in health care workers and Covid-19: A test negative case-control study. Vaccine.

[29] Ratnam S, West R, Gadag V, Williams B, Oates E (1997). Rubella antibody levels in school-aged children in Newfoundland: Implications for a two-dose rubella vaccination strategy. Can J Infect Dis..

